# Dataset for generating synthetic residential low-voltage grids in Sweden, Germany and the UK

**DOI:** 10.1016/j.dib.2021.107005

**Published:** 2021-04-06

**Authors:** Elias Hartvigsson, Mikael Odenberger, Peiyuan Chen, Emil Nyholm

**Affiliations:** a Department of Space Earth and the Environment, Division of Energy Technology, Chalmers University of Technology, Sweden; b Department of Electric Engineering, Division of Electric Power Engineering, Chalmers University of Technology, Sweden

**Keywords:** Reference network modeling, Residential solar PV, Grid capacity, GIS, low-voltage

## Abstract

Assessing grid capacity on national and local levels is important in order to formulate renewable energy targets, calculate integration costs of distributed generation (such as residential solar PV and electric vehicles). Currently, 70–96% of the residential solar PV installations in Germany and Italy are found in the low-voltage grid. Previous grid assessments have relied on grid data from individual low-voltage grids, making them limited to a few cases. This article presents synthetic low-voltage grid data from a reference network model. The reference network model generates synthetic low-voltage grids using publicly available data and national regulations and standards. In addition, the article presents data of residential solar photovoltaic hosting capacity in low-voltage grids. The datasets are high-resolution (1 × 1 km) and contains data on electricity peak demand, share of population living in apartments and important grid metrics such as transformer capacity, maximum feeder length and estimations of residential solar photovoltaic hosting capacity. Datasets on grid components are rare and the dataset can be used to assess grid impacts from other residential end-use technologies, and function as baseline for other reference network models.

## Specifications Table

**Table:** 

Subject	Electrical and Electronic engineering
Specific subject area	Electric power systems
Type of data	Figure
How data were acquired	Data was generated through reference network modeling. Software: MATLAB 2017b, Quantum GIS 3.6.3 (QGIS). Simulations were carried out on a workstation Intel Core i7 8700 (6 cores at 3.2 GHz) and 64GB of RAM.
Data format	Raw Analyzed
Parameters for data collection	For solar PV output: temperature, solar insolation and photovoltaic system orientation. Economic data: cost of power system components Technical data: capacity rating of power system components Regulations: regulations on voltage variation, tripping criteria and earth impedance.
Description of data collection	The data originates from simulations done in MATLAB. Primary datasets were processed in QGIS to achieve a resolution of 1 × 1 km before being loaded into MATLAB. In MATLAB each 1 × 1 km cell generates synthetic low-voltage grids based on dwelling distribution, population density, peak demand obtained from national specific sizing methods and low-voltage grid codes. The low-voltage grid codes consider voltage variation, tripping criteria, and thermal limits of components. Finally, earth impedance at the Point of Common Connection is compared with IEC data and adjusted accordingly. The generated synthetic low-voltage grids are then used to calculate residential solar photovoltaic hosting capacity.
Data source location	Primary data sources: Population density [1,2]: https://ec.europa.eu/eurostat/web/gisco/geodata/reference-data/population-distribution-demography/geostat https://www.scb.se/hitta-statistik/regional-statistik-och-kartor/geodata/oppna-geodata/statistik-pa-rutor/ Dwelling distribution [3] https://ec.europa.eu/CensusHub2 Earth impedance [4] https://webstore.iec.ch/publication/1865
Data accessibility	Data is hosted at the Mendeley Data repository http://dx.doi.org/10.17632/hn3ncrrj95.1 Code for generating data is available on Github http://dx.doi.org/10.5281/zenodo.4563951
Related research article	E. Hartvigsson, M. Odenberger, P. Chen, E. Nyholm, *Estimating national and local low-voltage grid capacity for residential solar photovoltaic in Sweden, UK and Germany*, Renewable Energy, https://doi.org/10.1016/j.renene.2021.02.073

## Value of the Data

• The dataset provides a baseline for future reference network modeling and load modeling, and it can be utilized to assess additional grid impacts of residential end-use technologies (such as electric vehicle charging, heat pumps) and by local and national governments as a tool for establishing technical targets. Open data from reference network modeling is rare, making the dataset especially useful.
• Amongst other, the dataset is useful for emerging researchers between energy system models and electric power systems. Previous, energy systems studies have generally focused on generation of electricity, partly due to a lack of data on grid capacity for new technologies. The attached dataset can help alleviate some of these issues, and allows for a better inclusions of grid capacity in energy system studies.
• The data represents different steps in reference network modeling for residential end-use purposes and can therefore be used at multiple steps for either validation or further development or more accurate reference network models.
• Grid integration costs are difficult to estimate, partly due to the variation in grid topology. The dataset can be used improve renewable energy grid integration in the low-voltage grids, and estimate and reduced grid integration costs.

## 1 Data Description

The following data is presented and available in cells with a geographical resolution of square km (sqkm), for Sweden, Germany and the United Kingdom (UK). The residential solar photovoltaic hosting capacity in the original research article is presented at a NUTS3/Local Administrative Units level [5]. The reason for this, as described in the sensitivity analysis in the original research article, is that the model's accuracy at the sqkm resolution is worse than at the NUTS3/Local Administrative Units level. Future use of the data with a sqkm resolution should take this into consideration.

**Fig. 1:**
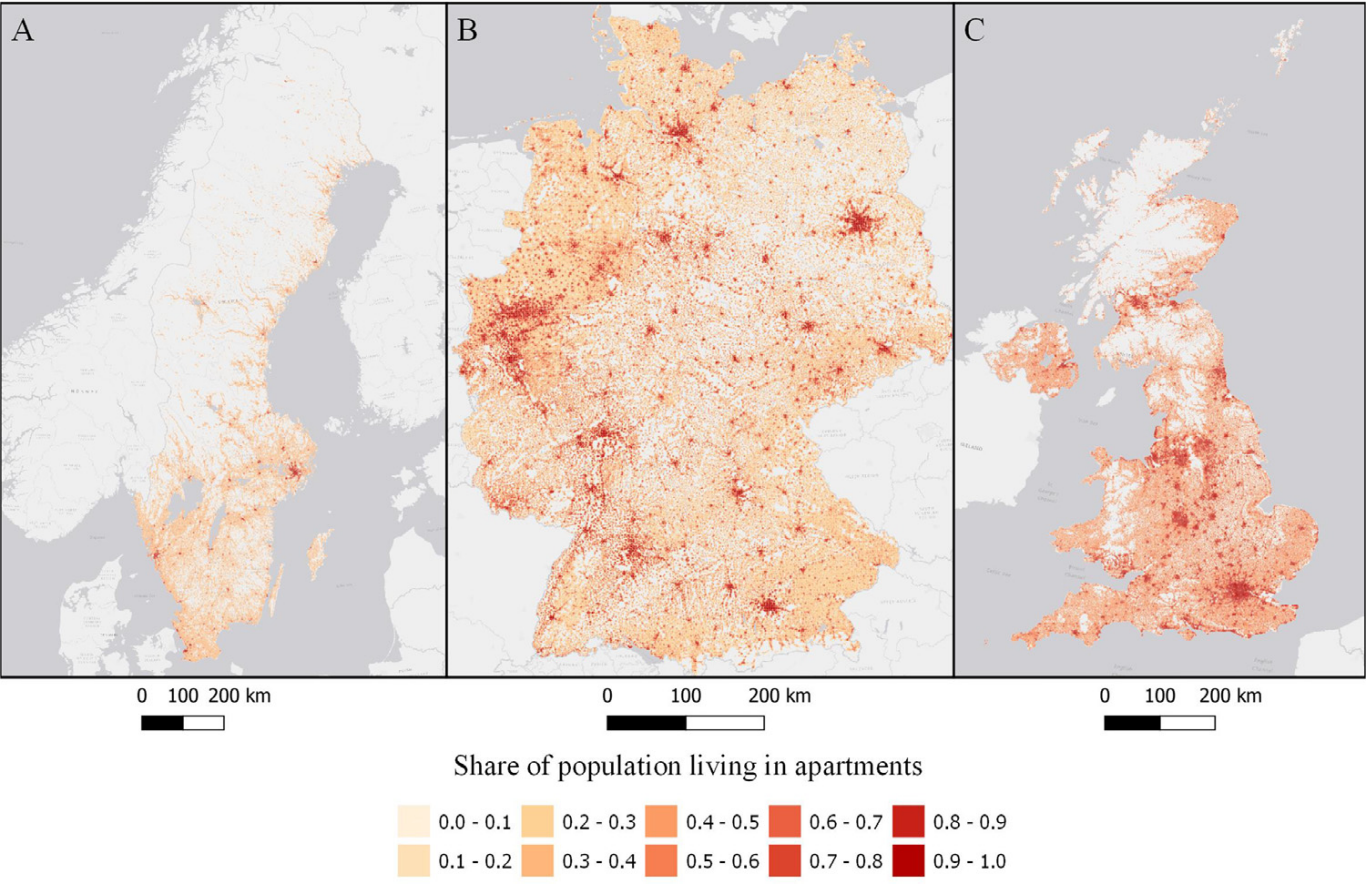
Share of population living in apartments in each cell (sqkm) for Sweden (A), Germany (B), and UK (C). ESRI Light gray canvas basemap [7].

**Fig. 2:**
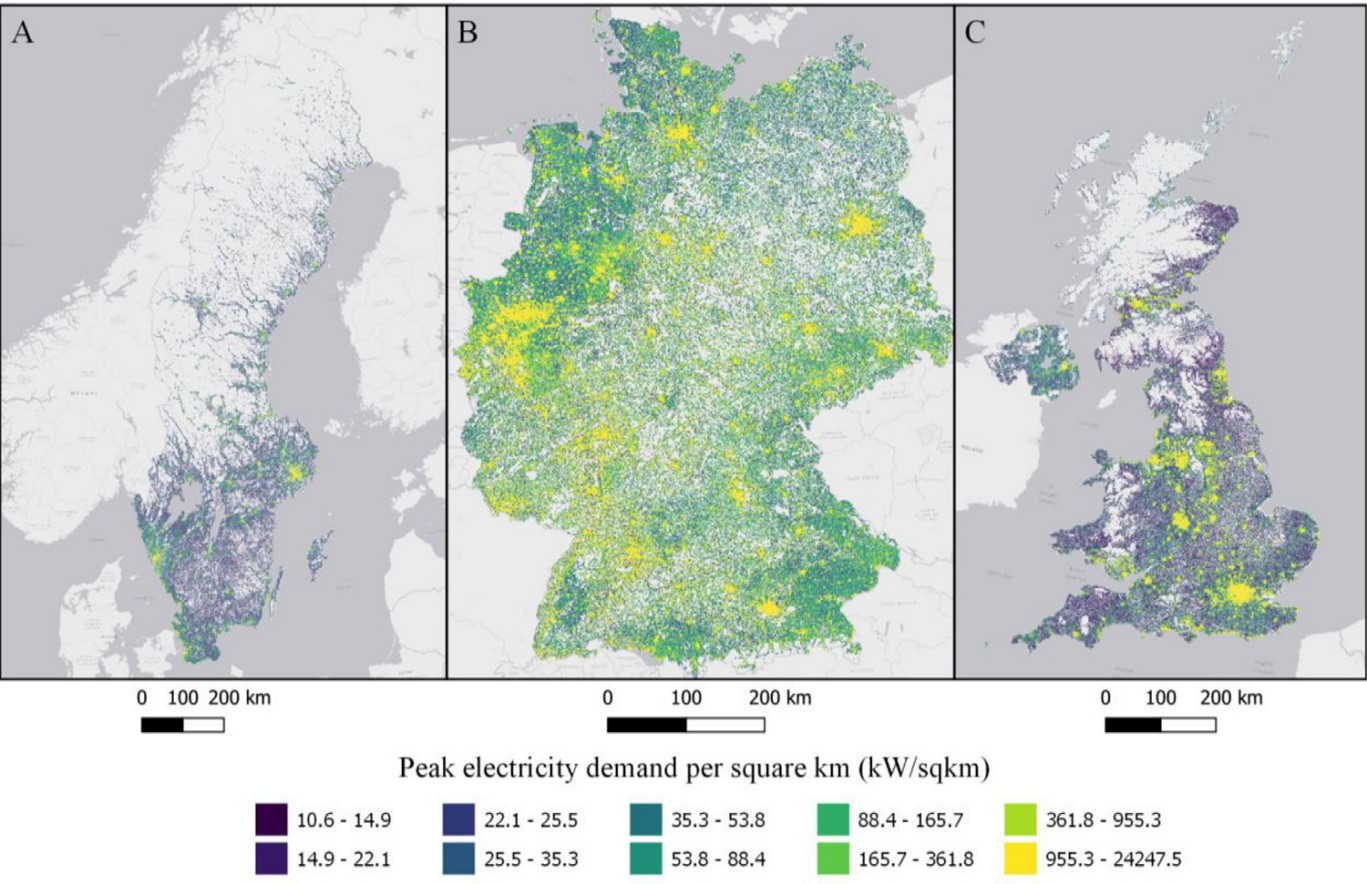
Residential peak load in each cell (kW/sqkm) in Sweden (A), Germany (B), and the UK (C). ESRI Light gray canvas basemap [7].

**Fig. 3:**
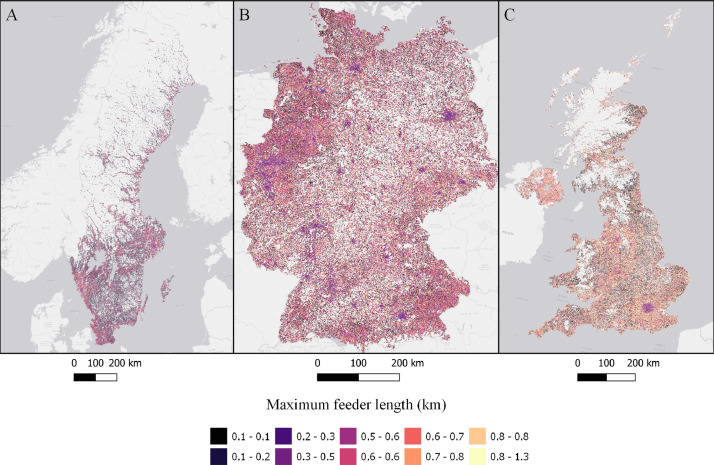
Longest low-voltage feeder per low-voltage grid in each cell (km) in Sweden (A), Germany (B), and the UK (C). ESRI Light gray canvas basemap [7].

**Fig. 4:**
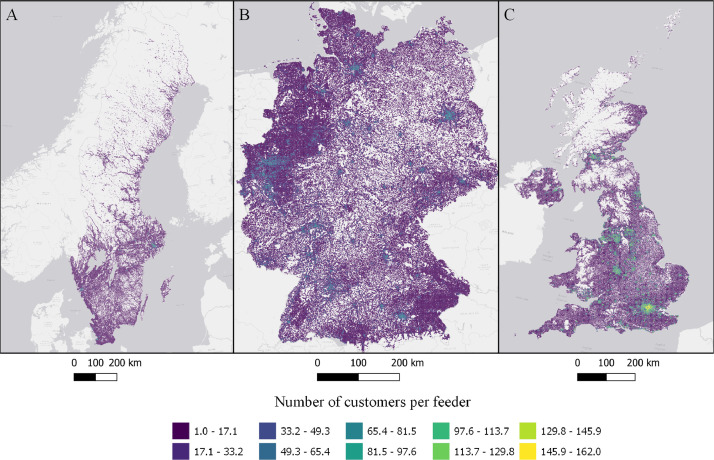
Number of customers connected to the longest feeder in each cell in Sweden (A), Germany (B), and the UK (C). ESRI Light gray canvas basemap [7].

**Fig. 5:**
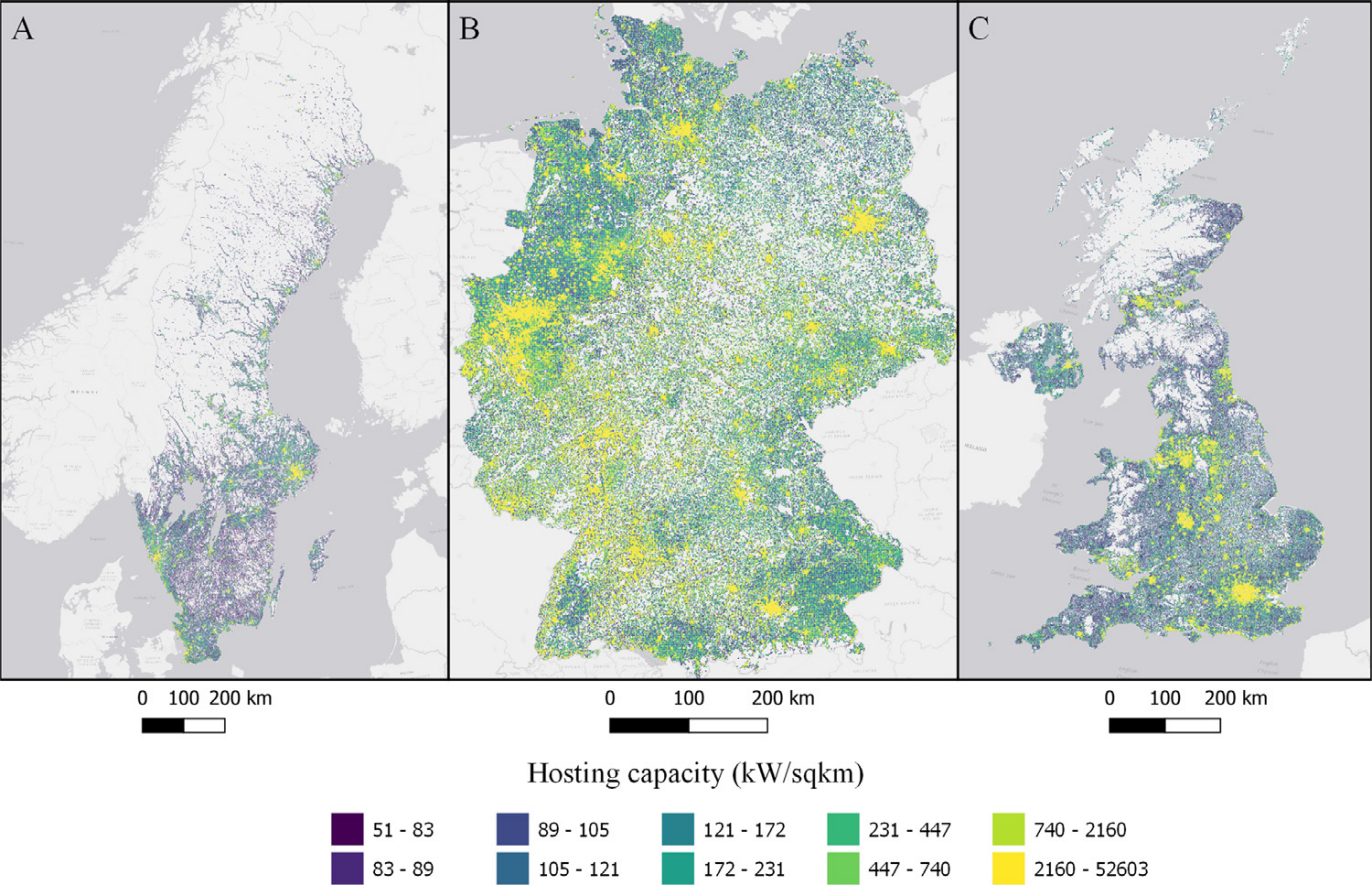
Hosting capacity in each cell (kW/sqkm) in Sweden (A), Germany (B), and the UK (C). ESRI Light gray canvas basemap [7].

**Fig. 6:**
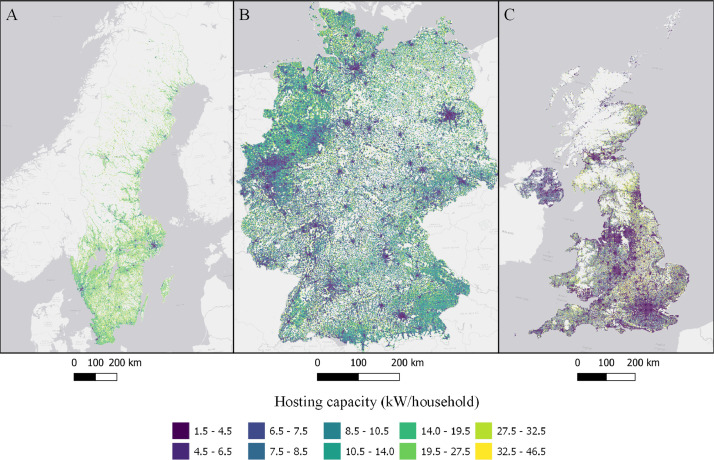
Hosting capacity expressed as capacity per household in each cell (kW/Household) in Sweden (A), Germany (B), and the UK (C). ESRI Light gray canvas basemap [7].

**Fig. 7:**
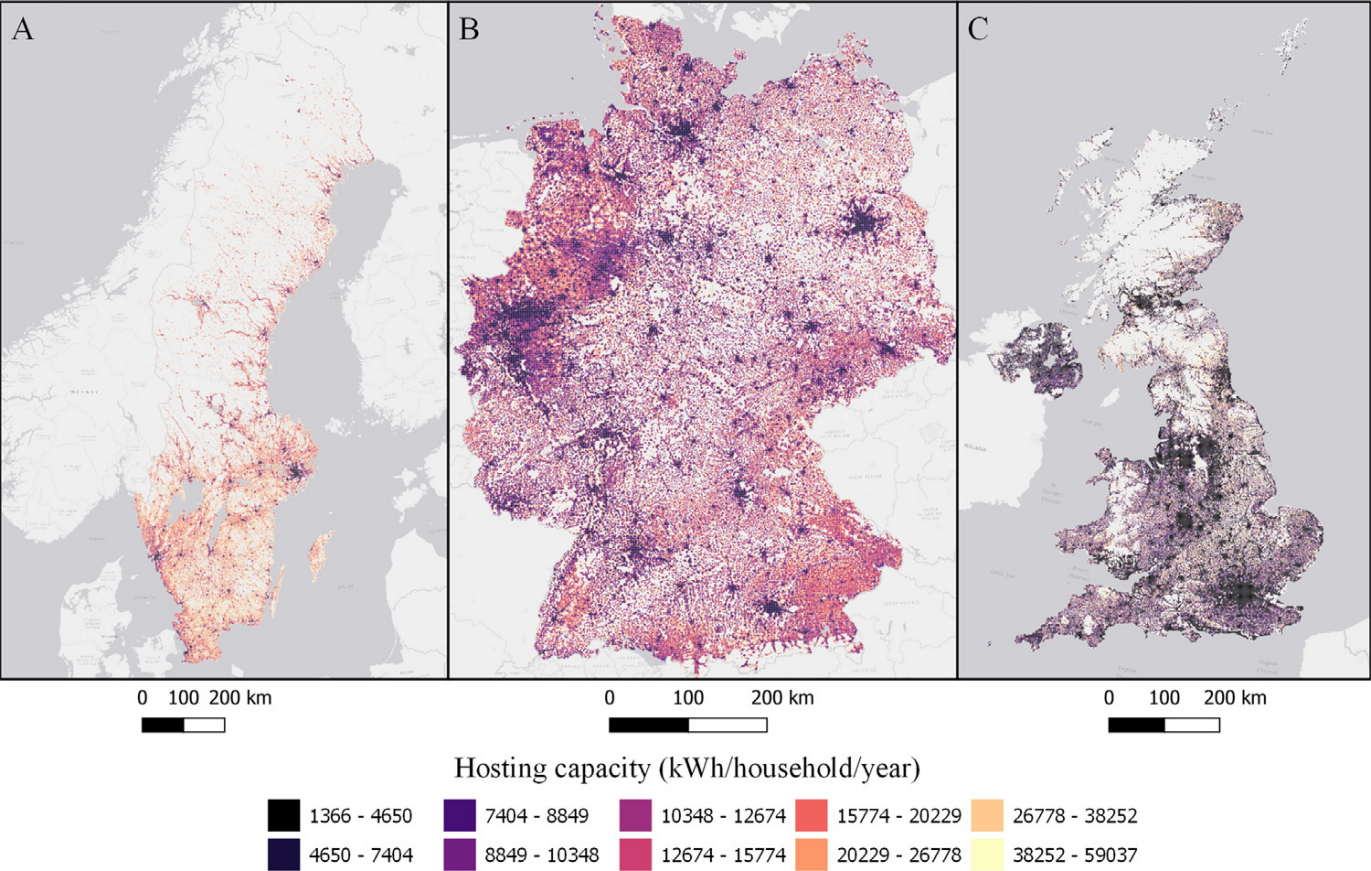
Hosting capacity expressed as annually produced electricity in each cell (kWh/household/year) in Sweden (A), Germany (B) and UK (C). ESRI Light gray canvas basemap [7].

**Fig. 8:**
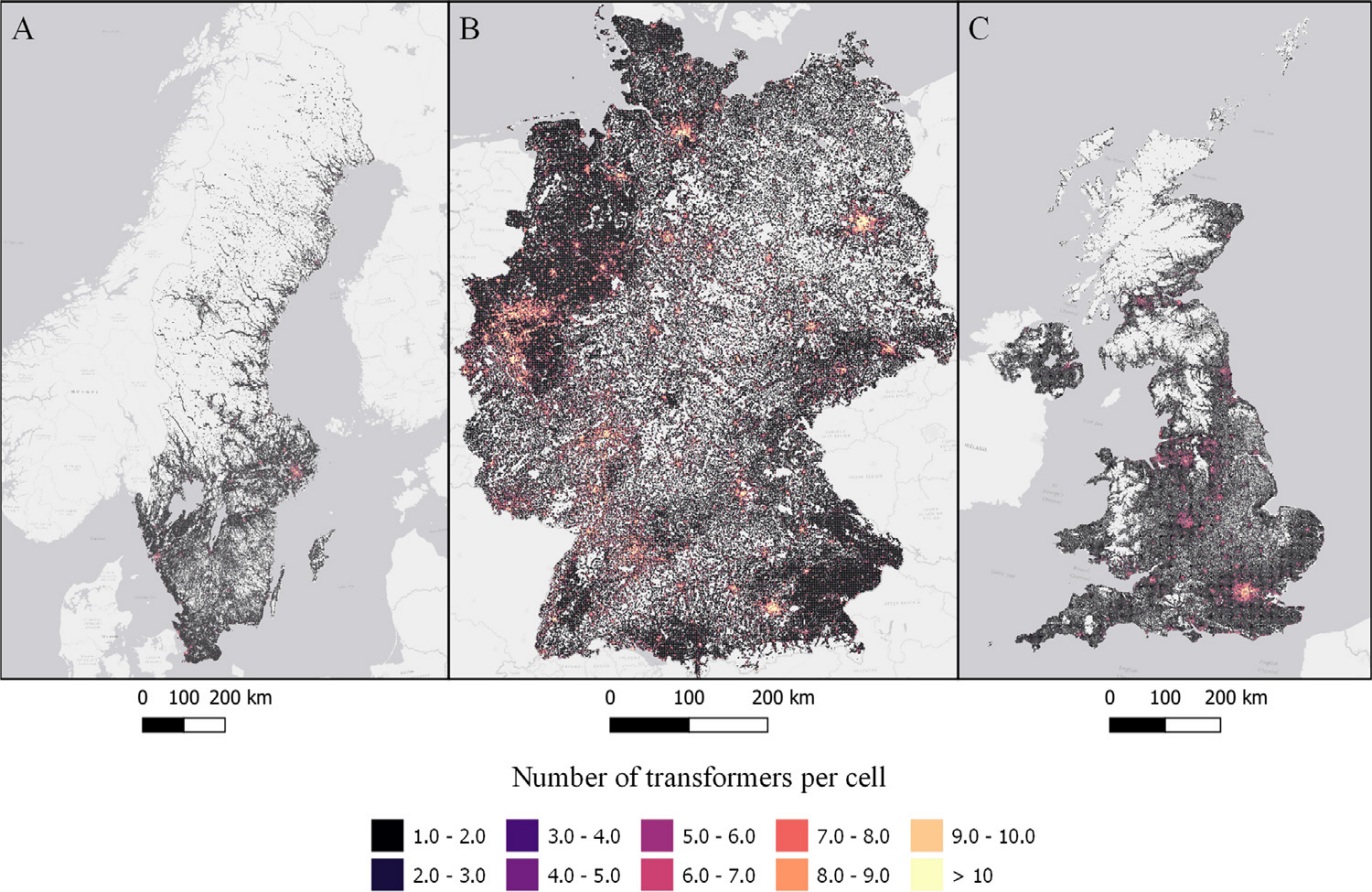
Number of transformers per cell (sqkm) in Sweden (A), Germany (B), and the UK (C). ESRI Light gray canvas basemap [7].

**Fig. 9:**
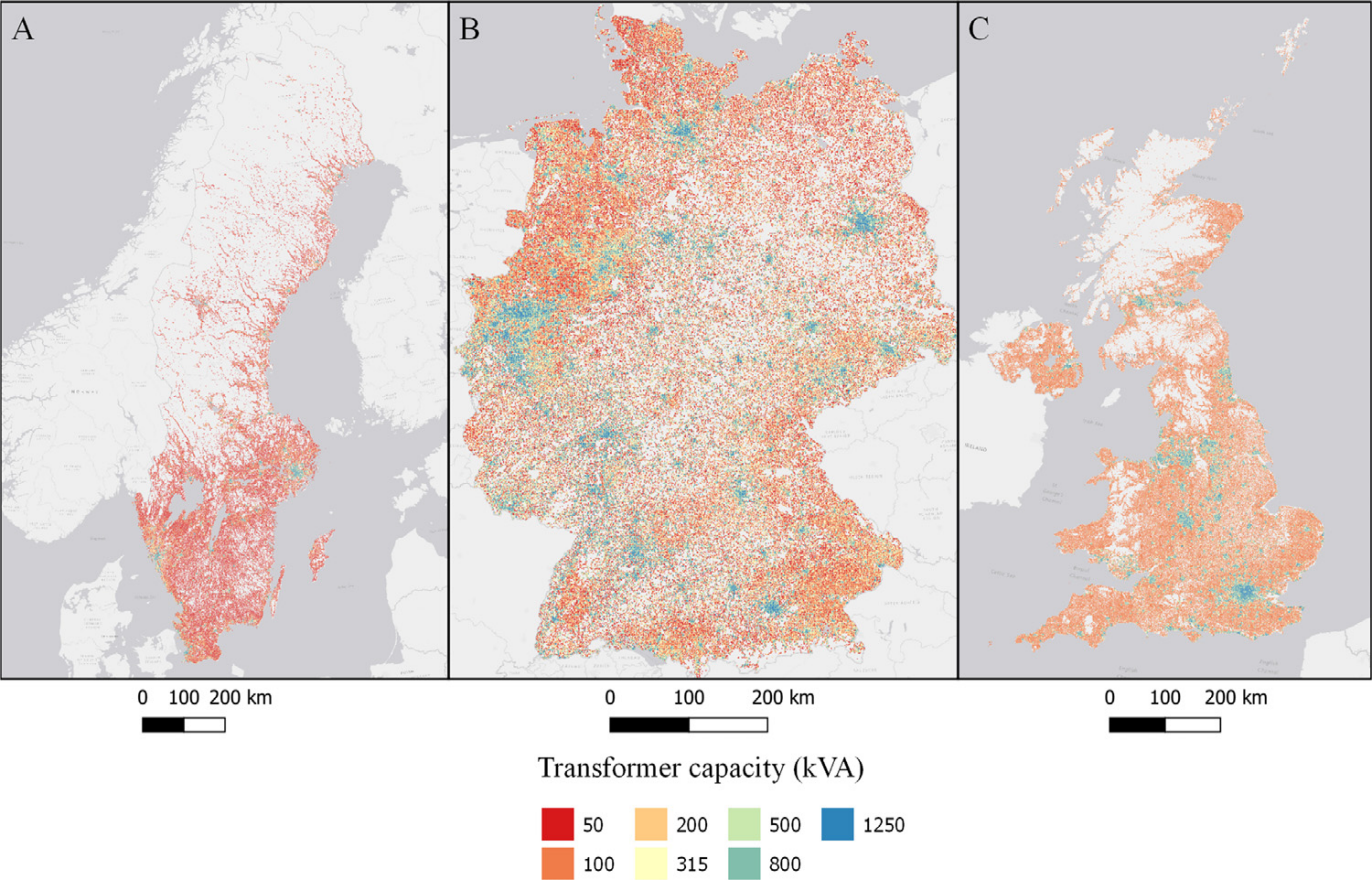
Transformer's capacity (kVA) in each cell in Sweden (A), Germany (B), and the UK (C). ESRI Light gray canvas basemap [7].

**Fig. 10:**
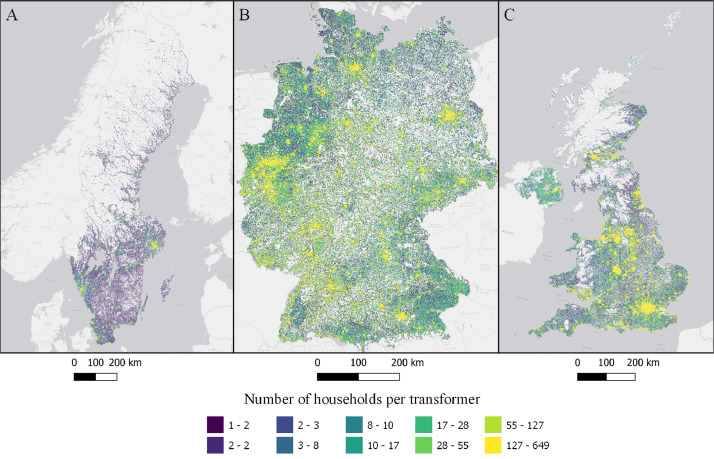
Number of households per transformer in each cell for Sweden (A), Germany (B) and UK (C). ESRI Light gray canvas basemap [7].

The data is available in the Mendeley Data portal [6] and is contained within three files **SWE_DataInBrief.zip** (Sweden), **UK_DataInBrief.zip** (UK) and **DE_DataInBrief.zip** (Germany). Each zip file contains all relevant GIS and data files for each country. All files use the EPSG:3035 (ETRS89/LAEA Europe) coordinate reference system. Figs. 1–10 below shows each dataset for the respective country. Table 1 include a list of all datasets, their corresponding variable names in the data files, and the Figure number.

**Table 1:** Datasets description, variable names and figure numbers.

Dataset	Variable name	Figure
Share of population living in apartments	FracAPT	1
Peak residential load demand (kW/sqkm)	Demand	2
Maximum feeder length (km)	Feeder	3
Number of customers on the longest feeder	CustPerFee	4
Hosting capacity (kW/sqkm)	Cap	5
Hosting capacity (kW/household)	CapPerCust	6
Hosting capacity (kWh/household)	EnergyHH	7
Number of transformers	TrNumber	8
Capacity of transformers (kVA)	TrCap	9
Household customers per transformer	CustPerTr	10

## 2 Experimental Design, Materials and Methods

A brief description of the method is presented here while a full, detailed description has been submitted to MethodsX. The computer code for the model is available on Github [8]. Hosting capacity for residential solar photovoltaic is calculated by generating synthetic low-voltage grids using public GIS data and national standards and regulations for low-voltage grids. The residential solar photovoltaic hosting capacity calculation are based on three steps: estimating peak electricity demand, generating synthetic low-voltage grids and calculating hosting capacity. Peak electricity demand is calculated using population density, share of population living in multifamily and single-family households and national specific power estimating methods. We use Velander's formula for Sweden, After Diversity Maximum Demand (ADMD) for the UK [9] (Eq. 2) and coincidence for Germany [10,11] for estimating the specific power. Share of population living in apartments are extrapolated from the 2011 EU census. The 2011 EU census contains data on NUTS3 or Local Administrative Units level. Depending on the country, the highest available geographical resolution is used. Using least square regression we identify the function with the best fit that models the share of population living in apartments as a function of population density.

Based on the calculated peak power demand, the number of transformers and their capacity is allocated using a cost-minimization strategy, were number and size of transformers are chosen to reduce total investment costs in a each cell. Hosting capacity is calculated using the longest feeder (continues stretch of cable or power line). The use of the longest feeder from each low-voltage grid was chosen due to improved computational feasibility. Conducting a power-flow analysis for each low-voltage grid would have significantly increased computational time making the problem computationally unfeasible for whole nations. Assuming a uniform distribution of customers, the maximum feeder length is calculated according to [12]. Cables are sized according to regulations and standards for voltage variation, tripping criteria and thermal capacity.

Hosting capacity calculations are sensitive to how solar PV systems are allocated in a low-voltage grid. Methods for allocation can be divided into stochastic and deterministic [13]. Stochastic methods randomly assign a location and size for a solar PV system given certain restrictions. Stochastic allocation method results in a solution space that contains a wide range of solar PV deployment scenarios but requires significantly more computing power. Due to the large geographical scope and to make the problem computational feasible we rely on a deterministic allocation method, were all solar PV systems are equally sized. Using the maximum feeder, the solar PV system sizes are simultaneously increased for all customers in steps of 0.5 kW until either the upper voltage level is reached, or the thermal limit of either the feeder or transformer is reached.

## CRediT Author Statement

**Elias Hartvigsson:** Conceptualization, Methodology, Software, Writing – original draft; **Mikael Odenberger:** Conceptualization, Writing – review & editing, Funding acquisition, Supervision; **Peiyuan Chen:** Conceptualization, Writing – review & editing, Supervision; **Emil Nyholm:** Methodology, Software, Writing – review & editing.

## Declaration of Competing Interest

The authors declare that they have no known competing financial interests or personal relationships that could have appeared to influence the work reported in this paper.
